# Grapefruit-Derived Micro and Nanovesicles Show Distinct Metabolome Profiles and Anticancer Activities in the A375 Human Melanoma Cell Line

**DOI:** 10.3390/cells9122722

**Published:** 2020-12-21

**Authors:** Christopher Stanly, Mariaevelina Alfieri, Alfredo Ambrosone, Antonietta Leone, Immacolata Fiume, Gabriella Pocsfalvi

**Affiliations:** 1 EVs-MS Research Group, Institute of Biosciences and BioResources, National Research Council, 80131 Naples, Italy; christopher.stanly@ibbr.cnr.it (C.S.); immacolata.fiume@ibbr.cnr.it (I.F.); 2 Department of Pharmacy, University of Salerno, 84084 Salerno, Italy; malfieri@unisa.it (M.A.); aambrosone@unisa.it (A.A.); aleone@unisa.it (A.L.)

**Keywords:** citrus, vesicles, nanovesicles, metabolome, antitumor activity, Akt signalling, ERK signalling, gas chromatography-mass spectrometry

## Abstract

Fruit juice is one of the most easily accessible resources for the isolation of plant-derived vesicles. Here we found that micro- and nano-sized vesicles (MVs and NVs) from four *Citrus* species, *C. sinensis*, *C. limon*, *C. paradisi* and *C. aurantium*, specifically inhibit the proliferation of lung, skin and breast cancer cells, with no substantial effect on the growth of non-cancer cells. Cellular and molecular analyses demonstrate that grapefruit-derived vesicles cause cell cycle arrest at G2/M checkpoint associated with a reduced cyclins B1 and B2 expression levels and the upregulation of cell cycle inhibitor p21. Further data suggest the inhibition of Akt and ERK signalling, reduced intercellular cell adhesion molecule-1 and cathepsins expressions, and the presence of cleaved PARP-1, all associated with the observed changes at the cellular level. Gas chromatography-mass spectrometry-based metabolomics reveals distinct metabolite profiles for the juice and vesicle fractions. NVs exhibit a high relative amount of amino acids and organic acids whereas MVs and fruit juice are characterized by a high percentage of sugars and sugar derivatives. Grapefruit-derived NVs are in particular rich in alpha–hydroxy acids and leucine/isoleucine, myo-inositol and doconexent, while quininic acid was detected in MVs. Our findings reveal the metabolite signatures of grapefruit-derived vesicles and substantiate their potential use in new anticancer strategies.

## 1. Introduction

There are several types of biomembrane-enclosed vesicles within and outside a cell that can transport or store bioactive molecules such as proteins, nucleic acids, lipids and other metabolites [1]. Secreted vesicles, called extracellular vesicles (EVs) are actively studied in mammalian systems due to their importance in cell-cell communication [2]. Mammalian EVs were shown to modulate the immune responses and play roles in physiological and pathological processes through the intercellular trafficking of functional molecules, including RNA, proteins and metabolites and are being exploited in both therapeutics and diagnostics [2]. EV-like vesicles have been isolated from apoplast and there are few pieces of evidence demonstrating their role in unconventional protein secretion, plant cell wall remodelling [2] and plant defence [3,4,5]. Moreover, apoplastic vesicles were shown to be involved in inter-kingdom communication by the intercellular transfer of small non-coding RNAs to fungal pathogens [3] and in long-distance gene regulations [6].

Recently, methods that conventionally are used to isolate EVs from mammalian resources have been employed to tissues, organs and juices of edible plant species [7], such as ginger and carrot [8], grape [9] and citrus fruits [10,11,12] to isolate EV-like vesicles. Studies show that the vesicle populations present in these isolates are very heterogeneous in size, morphology and origin. Intriguingly, edible plant-derived vesicles have good biocompatibility, high rate of cellular internalization and represent a valuable resource of several bioactive compounds, holding promise for nutraceutical and therapeutic purposes. For instance, vesicles extracted from grape were demonstrated to reach the intestinal mucus barrier, enter the mouse intestinal stem cells and stimulate tissue remodelling as well as providing protection against colitis in murine models [9]. Similarly, grapefruit-derived nanovesicles are specifically internalized by intestinal macrophages and alleviate drug-induced colitis in the mouse model [13] and vesicles extracted from lemon juice have interesting antineoplastic activities both in vitro and in vivo [11]. Several clinical trials are currently in progress to test the effectiveness of plant EVs in reducing insulin resistance, in controlling chronic inflammatory diseases (ClinicalTrials.gov Identifier: NCT03493984) [14] and in preventing some side effects of chemotherapy treatments (ClinicalTrials.gov identifier: NCT01668849) [15].

To improve our understanding of the biological activities of plant-derived vesicles it is important to know their molecular composition. In this regard proteins, RNA and lipids are the most studied and the metabolome, a set of small molecules found within or associated with the vesicles, is the least studied components of the biocargo. This component includes chemically diverse molecules with different lipophilicity, polarity and stability. Metabolites are usually classified based on their functional groups. Low-molecular-weight metabolites (below 900 Da molecular mass) include aliphatics, alcohols, amides, amino acids, carboxylic acids, and sugars [16]. Lipids and their derivatives are also metabolites but usually considered separately in EV analysis. The relationship between the metabolome and bioactivity of vesicles is of particular interest and can give rise of novel studies including precision nutrition guided by metabolomics [17].

Citrus fruits, including oranges, lemons and grapefruits, are consumed worldwide either as fresh fruits or in the form of juices. In particular, the consumption of citrus fruits and juice is highly recommended owing to the elevated contents of vitamins, minerals, fibre, and phytochemicals with health benefits, such as carotenoids, flavonoids, and limonoids, with antioxidant, anti-inflammatory and antitumor activities [18]. Citrus fruits and their extracts also provide a blend of natural compounds, which are increasingly employed by the cosmetic and pharmaceutical industries. Considering these benefits, it is of great interest to gain a deeper understanding of vesicles that can be isolated from the juice of citrus fruits and to examine their potential use for human health. We have recently published data on the isolation and physicochemical and protein characterization of vesicles derived from four *Citrus* species, namely orange (*C. sinensis*), bitter orange (*C. aurantium*), lemon (*C. limon*) and grapefruit (*C. paradisi*) [12]. Vesicle populations present in the juice of these citrus species could be separated into microvesicles rich (MVs between 350 and 700 nm in diameter) and nanovesicles rich (NVs between 50 and 80 nm in diameter) fractions using differential ultracentrifugation. Proteome analyses revealed the presence of approximately 600–800 proteins in each sample including many hydrolases, oxidoreductases and antioxidant enzymes such as catalase, superoxide dismutase, peroxidase, ascorbate peroxidase and glutathione reductase [12].

Here, citrus-derived vesicles were screened for their antitumor activities on different cell lines. The best performing candidates were further investigated at the cellular and molecular levels on a melanoma cancer cell line and regarding their metabolites profiles of small molecules to identify molecules with potential anti-tumour activity. Our findings highlight the importance of citrus-derived nano- and microvesicles as natural carriers of bioactive compounds, suggesting their role in the prevention of cancer as well as their possible use as therapeutic adjuvants in cancer treatments.

## 2. Materials and Methods

### 2.1. Plant Material and Vesicle Isolation

Fruits of four different *Citrus* species, sweet orange (*C. sinensis*), lemon (*C. limon*), grapefruit (*C. paradisi*) and bitter orange (*C. aurantium*) grown without any pre- and postharvest treatments were collected at maturity stage 3 from local gardens in Naples, Italy. Approximately 500 mL of fresh fruit juice was obtained by squeezing from five to ten pieces of fruits. Protease inhibitor cocktail, containing complete ultra tablets EDTA free (Roche, Mannheim, Germany) prepared according to the manufacturer’s instruction, Leupeptine (0.25 mL, 1 mg/mL), Phenylmethylsulfonyl fluoride (PMSF) (1.25 mL, 100 mM) and sodium azide (0.8 mL, 1 M) were added to each sample. Vesicles were isolated in four parallel experiments by differential ultracentrifugation, following the procedure described by Stanly et al. [1]. Briefly, low-velocity centrifugation steps were performed at 400× *g* and 1000× *g* for 20 min at 22 °C to eliminate cell debris and at 15,000× *g* for 20 min at 22 °C to collect the fraction enriched in MVs. The supernatant was ultracentrifuged at 150,000× *g* for 60 min at 4 °C using a 70Ti Beckman rotor and the resulting pellet was used for the purification of NVs. To remove co-purifying pectins, pellets obtained after the 15,000× *g* and 150,000× *g* centrifugation steps were solubilized in 50 mM Tris-HCl pH 8.6 and re-centrifuged two more times using the same centrifugal conditions. Finally, the pellet was solubilized in PBS buffer. Protein concentrations were measured by micro BCA assay (Thermo Scientific, Rockford, IL, USA), using Nanodrop 2000 (Thermo Fisher Scientific Inc., Waltham, MA, USA). Samples were used fresh for subsequent characterization and the remaining samples were conserved at −80 °C.

### 2.2. Metabolite Extraction and Derivatization

Metabolites from fruit juice were extracted by the phase separation method according to Roessner et al. [19]. Briefly, 200 μL filtered juice was mixed with 600 μL methanol, 200 μL chloroform, and 10 μL of 10 mg·mL^−1^ Nor leucine (Sigma Aldrich, St. Louis, MO, USA) solution in water as an internal standard and vortexed for 5 min. 400 μL of water was added, mixed well and centrifuged at 10,000× *g* for 5 min to separate the polar and nonpolar phases. Two-hundred microlitres (200 μL) of the upper polar phase was used for derivatization reaction.

Metabolites from MV and NV fractions were extracted as follows: 200 μg (expressed in protein content measured by the micro BCA assay) of vesicles were pelleted at 15,000× *g* for 30 min for MVs and 110,000× *g* for 1 h for NVs. The vesicle containing pellets were solubilized in 1 mL of methanol:water: chloroform, 2.5:1:1 (*v*/*v*/*v*) to which 150 nmol of Norleucine internal standard was added, mixed well and centrifuged at 16,000× *g* for 30 min. The supernatant was transferred to a new 2 mL Eppendorf tube. 0.5 mL of methanol: chloroform, 1:1 (*v*/*v*) was added to the pellet mixed well and centrifuged at 16,000× *g* for 30 min. The two supernatants were combined and 300 μL water was added and the sample was centrifuged at 16,000× *g* for 30 min. The upper polar phase was transferred to a new tube and dried in a vacuum drier.

The trimethylsilyl (TMS) derivatization was performed by dissolving the dried extracts in 50 μL of methoxyamine hydrochloride (MOX, Sigma Aldrich, St. Louis, MO, USA) solution (in pyridine 20 mg/mL, *w*/*v*), rigorously mixed (vortexed). MOX reacts with both aldehydes and ketones to form oxim methyl ethers and thus prevents multiple derivatives when enols are present during silylation. The reaction was performed at 30 °C for 90 min. Trimethylsilylation was performed by adding 50 μL of N, O-Bis(trimethylsilyl)trifluoroacetamide (BSTFA) and trimethylchlorosilane (TMCS) 99:1 (Supelco, Sigma-Aldrich, St. Louis, MO, USA) to the samples, mixing and incubating at 37 °C for 60 min.

### 2.3. GC-MS Analysis and Data Elaboration

The Trace 1300 GC coupled to the TSQ DUO triple quadrupole mass spectrometer (Thermo Scientific, Waltham, MA, USA) was used for GC-MS analysis, The derivatized samples (1 μL) were separated using a DB-5 column (30 m length, 0.25 mm internal diameter, 0.25 m film, Thermo Scientific, Waltham, MA, USA). The GC oven conditions were as follows: initial temperature 70 °C hold 1 min, ramp-1 1 °C/min to 76 °C, ramp-2 6 °C/min to 200 °C, ramp-3 40 °C/min to 325 °C, and hold 5 min. Helium was used as a carrier gas at a 1.2 mL/min constant flow. The total run time was 40 min. The transfer line and ion source temperatures were 240 and 250 °C, respectively. Electron impact ionization was used at 70 eV. EI mass spectra were acquired using full scan mode. Acquisitions were performed in the range of m/z 40–600, with a scan time 250 ms. The analysis was carried out in triplicate. The identification of each of the metabolites was conducted using a Thermo Scientific Chromeleon Chromatography Data System (CDS) (Thermo Fisher Scientific, Inc.). Compounds were identified through mass spectral matching using the NIST database and applying a match cut-off criteria of 700/1000.

### 2.4. Cell Cultures

Breast adenocarcinoma (MCF7), human melanoma (A375), lung adenocarcinoma (A549) and human normal skin keratinocyte (HaCat) cells, were maintained in Dulbecco’s modified Eagle medium (DMEM), supplemented with 10% (*v*/*v*) FBS, 2 mM L-glutamine and antibiotics (100 U mL^−1^ penicillin, 100 μg mL^−1^ streptomycin) from Invitrogen (Carlsbad, CA, USA), at 37 °C in a humidified atmosphere with 5% CO_2_.

### 2.5. Cell Proliferation and Viability

MCF7, A549, A375 and HaCat cells (approximatively 5000/well) were seeded in triplicate in 96 well-plates and incubated for 24 h and 48 h with the different citrus-derived NVs or MVs at a concentration of 2.5 μg mL^−1^. The number of viable cells was quantified by [3-4,5-dimethyldiazol-2-yl]- 2,5-diphenyl tetrazolium bromide (MTT) conversion assay (Sigma-Aldrich, St. Louis, MO, USA). Following the vesicles treatment, cells were incubated with MTT (5 mg/mL in PBS) for an additional 2 h at 37 °C. Thereafter, cells were lysed and solubilized with 100 μL of buffer containing 50% (*v*:*v*) N, N-dimethylformamide, 20% SDS, with an adjusted pH 4.5 (Opipari et al. 1992). The absorbance was measured at 570 nm with a microplate reader (Titertek multiskan MCC7340, LabSystems, Vienna, VA, USA).

### 2.6. Analysis of Cell Cycle by Flow Cytometry

DNA content was measured by propidium iodide (PI) incorporation into permeabilized cells, as described by Nicoletti et al. [20]. Briefly, cancer cells were harvested after treatment with NVs or MVs and incubated with a PI solution (0.1% sodium citrate, 0.1% Triton X-100 and 50 μg mL^−1^ of PI (Sigma-Aldrich, St. Louis, MO, USA), 10 μg mL^−1^ RNAse A for 30 min at room temperature. Data from 10,000 events for each sample were collected by a FACS Calibur flow cytometer (Becton–Dickinson, San Josè, CA, USA). The percentage of cells in the different cell cycle phases was determinate using the DNA analysis ModFit LT^TM^ software (Verity Software House, Topsham, ME, USA). Data were expressed as the mean ± SD of three experiments, each performed in triplicate.

### 2.7. Western Blotting

Cells were lysed in a buffer containing 10 mM Tris HCl, pH 7.5, NaCl 150 mM, and Triton 1% supplemented with Protease Inhibitor Cocktails (Sigma Aldrich, Saint Louis, MO, USA) and the protein content was measured by a colourimetric assay (BIO-RAD, Hercules, CA, USA). Protein cell lysates (20 μg) were separated by SDS-PAGE under denaturing conditions, transferred onto a PVDF filter (BIO-RAD, Hercules, CA, USA), blocked with 5% milk and probed with primary antibodies: anti-ERK2, anti-phospho-ERK, anti-ICAM1, anti-PARP1, anti-pan-cathepsin and anti-GAPDH (Santa Cruz Biotechnology, Dallas, TX, USA), and anti-AKT and anti-phospho-AKT (Ser-473) (Cell Signalling Technologies, Danvers, MA, USA). After primary antibody incubation, the blots were washed and incubated with horseradish peroxidase-conjugated secondary antibodies (ThermoFisher Scientific; Waltham, MA, USA). The proteins recognized by Abs were detected by ECL (Amersham biosciences; Little Chalfont, UK). Pre-stained protein markers (ThermoFisher Scientific; Waltham, MA, USA) were used as molecular size standards. All experiments were performed in triplicate.

### 2.8. RNA Extraction and qRT-PCR Analyses

Total RNA from A375 cells was extracted by using TRIzol reagent (Invitrogen, Carlsbad, CA, USA), according to the manufacturer’s instructions. Complementary DNA (cDNA) was synthesized from 1 μg total RNA, previously treated with RNase-free DNAse I, using random hexamers and the Superscript III RT (Invitrogen, Carlsbad, CA, USA) at 50 °C for 50 min. Real-time quantitative reverse transcription PCR (qRT-PCR) reactions were performed in a 20 μL volume, consisting of 1× Cycler-DNA Master SYBR Green I mix (Roche Diagnostics Ltd., Lewes, UK), several cDNA dilutions and 0.5 μM each primer. The reactions were run in a LightCycler 480 rapid thermal cycler system (Roche Diagnostics Ltd., Lewes, UK) under the following fast-cycling steps: initial denaturation for 2 min at 95 °C, followed by 40 cycles at 95 °C for 2 s, 58 °C for 20 s and 72 °C for 5 s.

Also, melting curves (20 min; from 58 °C to 90 °C) were generated to check any spurious amplification products. To normalize RNA levels, the *GAPDH* (NM_002046.7) housekeeping gene was used as a reference control. The sequences of all the primers used are listed in Table S1.

Three technical repeats from three independent biological experiments were carried out. The delta-delta Ct (2^−ΔΔCT^) method, for comparing relative expression results between treatments, was applied [21].

## 3. Results

### 3.1. Impact of Citrus-Derived Vesicles on Tumour Cell Lines

**Figure 1:**
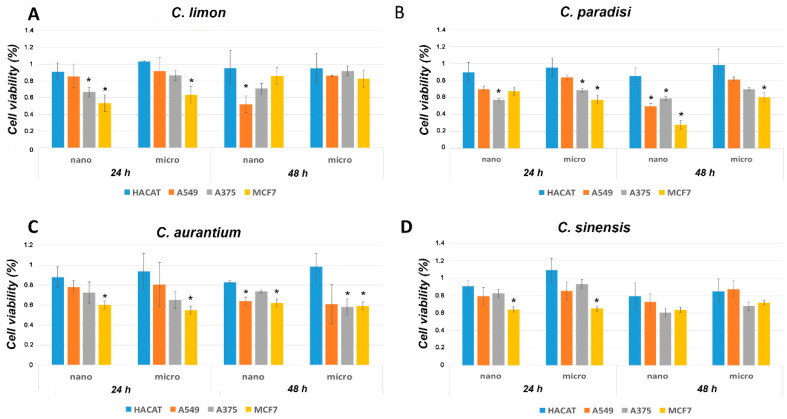
Citrus-derived nano and microvesicles reduce cancer cell proliferation. Cell growth was measured by MTT assay after 24 h and 48 h incubation with 25 μg/mL (expressed in protein content) of nano- and microvesicles isolated from the fruit juices of the following *Citrus* species: (**A**) *C. limon,* (**B**) *C. paradisi*, (**C**) *C aurantium* and (**D**) *C. sinensis*. The values were plotted as fold changes concerning related mock-treated cells (100% viable). Each point represents the mean ± SD of three independent experiments. Asterisks denote statistically significant values in comparison to non-tumour cells (HaCaT) assessed by *t*-test (* *p* ≤ 0.05).

MVs and NVs isolated from four different *Citrus* species (*C. sinensis*, *C. aurantium*, *C. limon* and *C. paradisi*) were evaluated for their ability to influence the growth of HaCaT (human keratinocytes), A375 (melanoma), A549 (lung adenocarcinoma) and MCF-7 (breast carcinoma) cell lines. Cells were incubated for 24 h and 48 h with 25 μg/mL of NVs or MVs (expressed as protein content) and cell viability was determined by the tetrazolium-based cytotoxicity assay (MTT). As shown in Figure 1, non-cancer cells treated with MVs and NVs isolated from four different *Citrus* species did not alter HaCaT viability suggesting a safe profile for normal cell lines. Conversely, all citrus-derived vesicles, although to a different extent, affected negatively the cell viability of different tumour cell lines, thus suggesting specific bioactivity towards cancer cells.

Moreover, data indicate that NVs and MVs show comparable effects among the single *Citrus* species, with some exceptions. Indeed, lemon-derived NVs severely inhibited the viability of MCF7 and A549 cells after 24 h and 48 h (Figure 1A), respectively, thus showing higher bioactivity with the respect to lemon-derived MVs. As for grapefruit, NVs reduced more than 40% the viability of all cancer cells showing the most pronounced effects among the vesicles investigated in this work. Grapefruit-derived vesicles according to this experimental finding and previous data show a good anti-cancer therapeutic potential [13]. NVs and MVs derived from grapefruit thus were further investigated for their antitumour activity.

Among the cell lines used in the bioactivity screening, A375 and MCF7 were the most responsive showing more than 30% viability reduction in several treatments. In this work, given their early significant responsiveness (24 h) to both NVs and MVs treatments, we selected A375 cell line as a model for further experiments aiming at studying the interactions between grapefruit-derived EV-like vesicles and human malignant melanoma cells.

**Figure 2:**
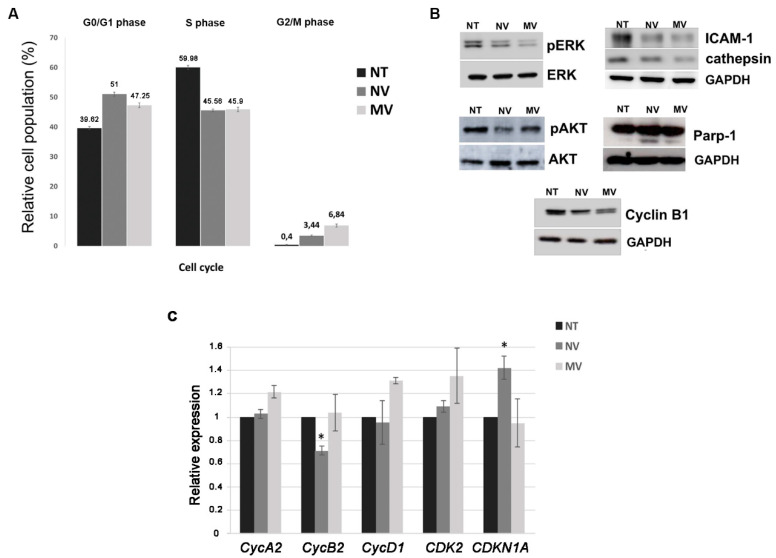
Grapefruit-derived vesicles induce cell cycle arrest and affect the expression of oncogenes and cell-cycle regulatory genes in melanoma cells (**A**) Flow cytometry analysis of melanoma A375 cells untreated (NT) or treated with 25 μg/mL nanovesicles (NVs) or microvesicles (MVs) for 24 h. Cell distribution in the different cell cycle phases was determined by propidium iodide staining. Each point represents the mean ± SD of three independent experiments. (**B**) Western blot analyses were performed on cells treated for 24 h with 25 μg/mL of *C. paradisi*-derived nanovesicles or microvesicles. Levels of proteins involved in cell proliferation (pERK and pAkt), oncogenes (ICAM 1 and cathepsin) and apoptosis (Parp-1) were evaluated. Blots were stripped and subsequently re-probed with an antibody against GAPDH to check equal loading of protein extracts. (**C**) Relative expression levels of cell-cycle regulatory genes in 24 h NV- and MV-treated melanoma cells by qRT-PCR. *GAPDH* was used as a reference gene to normalize the expression levels. Data represent mean ± SD of three technical repeats from three biological replicates, expressed as relative fold changes compared to their basal level in untreated A375 cells. Statistically significant values in untreated A375 cells assessed by t-test (* *p* ≤ 0.05).

To investigate the effects on tumour cell cycle, A375 cells exposed to grapefruit-derived MVs and NVs were analysed by FACS. We noticed an increase in the number of cells in the G0/G1 phase and consequently a decrease in the number of cells in S phase at 24h. Additionally, we also found that vesicles promote a significant enrichment of cell population in the G2/M phase, indicating that the cell cycle at the G2/M checkpoint was arrested as well (Figure 2A). Levels of cyclin B, which is critically involved in the control of cell cycle progression from G2 to M phase [22] were measured to correlate with the observed checkpoint arrest. In accordance with the FACS results, we have found that 24 h vesicles treatment lowers the protein levels of cyclin B (Figure 2B). To provide further insights into the effects of MVs and NVs on the cell cycle, the expression levels of three cyclins (*CycA2*, *CycB2*, *CycD1*), a cyclin-dependent kinase (*CDK2*) and a cyclin-dependent kinase inhibitor 1 (*CDKN1,* also known as *p21*) were studied in A375 cells treated with 25 μg/mL NVs and MVs (Figure 2C). Interestingly, qRT-PCR analyses revealed that NVs knockdown the expression of *CycB2*, an essential regulator of G2/M transition. CycB2 is commonly overexpressed in many malignant tumours and its down-regulation impairs cell invasion and metastatic abilities [23]. We also observed a significant up-regulation of *CDKN1*, which may contribute to reinforcing the cell cycle arrest. Indeed *CDKN1* is a potent cyclin-dependent kinase inhibitor playing a pivotal role in cancer suppression and therapy [24]. Collectively, these data provide convincing evidence that vesicles obtained from grapefruit promote cell cycles arrests, in part associated with cyclins B1 and B2 downregulation and *CDKN1* upregulation, and ultimately impair the growth of melanoma cells in vitro.

Since our data showed that NVs and MVs from grapefruit reduced cycling and viability of cancer cells, we sought to assess their effects on the regulation of key molecular cascades involved in cancer cell survival, proliferation and aggressiveness, such as the phosphatidylinositol 3-kinase (PI3K)/AKT pathway and mitogen-activated protein kinase (MAPK)/extracellular signal-regulated kinase (ERK) pathway. PI3K/AKT and MAPK/ERK pathways are aberrantly regulated in almost one-third of human cancers, and often both pathways are concurrently activated, thus representing excellent targets for cancer therapy [9,25]. Malignant melanomas have elevated AKT phosphorylation or activated PI3K/AKT/mTOR pathways [26,27,28]. Similarly, the high prevalence of activating mutations of ERK-MAPK signalling in a large fraction of human melanoma tumours supports its critical role in this pathogenesis [29,30]. Targeting these signal transduction pathways holds great therapeutic promise to arrest melanoma progression [31,32,33,34]. A375 cells were incubated for 24 h with 25 μg/mL vesicles and the protein levels of Akt, phospho-Akt (p-AKT), ERK1/2 and phospho-ERK1/2 (p-ERK) were assessed by Western blotting. In particular, Figure 2B shows that both NVs and MVs suppress phosphorylation of ERK and Akt, while no obvious changes of ERK and Akt protein levels were detected. Taken together, these data suggest that MVs and NVs isolated from the fruit juice of *C. paradisi* inhibit Akt and ERK signalling, thus impairing cancer cell viability. Furthermore, we explored the pro-apoptotic activity of grapefruit vesicles by investigating the cleavage of poly (ADP-ribose) polymerase-1 (PARP-1). Western blotting in Figure 2B shows the presence of the cleaved PARP-1 both in NVs and MVs treated A375 cells.

Melanoma is one of the most aggressive cancers with high metastatic potential. Therefore, to examine the impact of vesicles isolated from grapefruit on tumour cell aggressiveness, we evaluated the expression of cysteine protease cathepsins and intercellular adhesion molecule 1 (ICAM), two important markers of the tumour spreading. In particular, ICAM-1 upregulation is required to initiate the lymphatic spread of melanoma [34] while different cathepsins have been demonstrated to be up-regulated in most primary melanomas and all cutaneous melanoma metastases. Interestingly, we found that grapefruit-derived NVs and MVs reduce ICAM 1 expression, possibly limiting the uncontrolled adhesion capability of melanoma cells. Similarly, incubation with grapefruit vesicles also lowers the expression of cathepsins in A375 cells, thus reducing their capability to invade surrounding tissues. Altogether, these data suggest that grapefruit-derived vesicles may mitigate the invasiveness of melanoma holding great promise to control the metastatic potential in cancer therapy.

### 3.2. Metabolome Profiles of Grapefruit-Derived Micro and Nanovesicles

**Figure 3:**
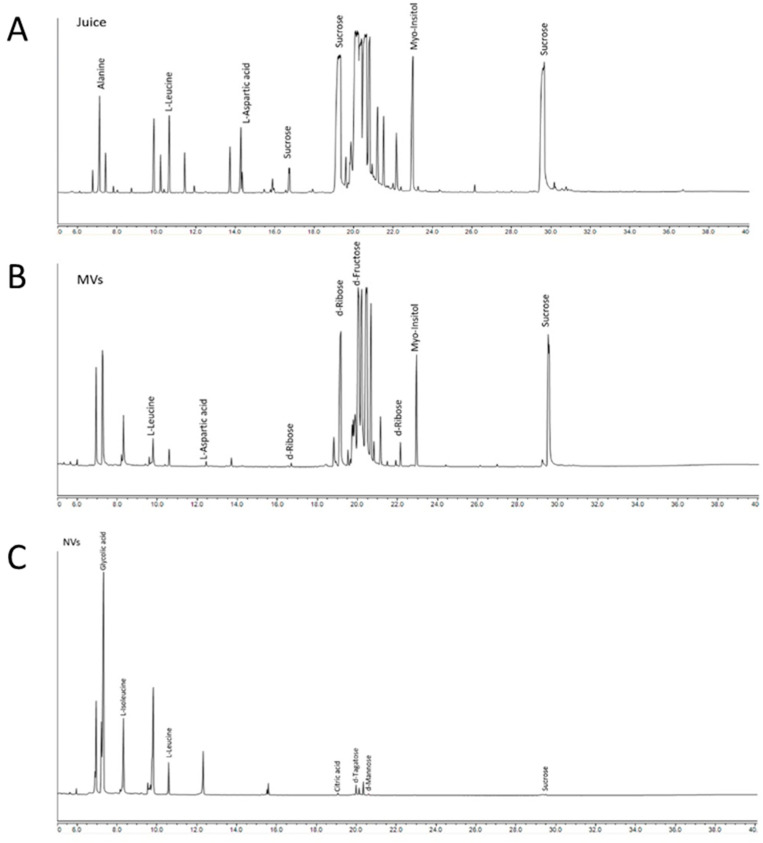
Metabolite profiles of (**A**) whole juice, (**B**) microvesicles and (**C**) nanovesicles isolated from grapefruit and analysed by GC-MS in full scan mode. Chromatograms are showing the most abundant GC-MS peaks and their tentative identifications.

Polar metabolites from juice, MVs and NVs samples isolated from grapefruit were extracted by chloroform/methanol/water partitioning using phase separation and analysed by GC-MS to reveal metabolome profiles. GC-MS is a highly sensitive and high-throughput analytical technique, useful for targeted and untargeted studies of metabolites in plants. Here, we performed an untargeted GC-MS study to analyze and tentatively identify the extracted metabolites and to obtain the concentration profiles. More than 100 peaks were detected in each sample of juice and MVs and about 80 in NVs from which about one third were tentatively identified by spectral matching in the NIST reference library (Table S2). Typical GC-MS profiles obtained by full scan mode are shown in Figure 3.

Fructose is the most abundant metabolite detected in grapefruit juice followed by citric acid, glucose, sucrose and myo-inositol (Table 1A). Similarly, we have found high amounts of mono- and disaccharides and derivatives in MVs (Table 1B). For example, myo-inositol a well-known carbocyclic sugar that is widely used in health-promoting supplement formulations and abundant in fresh fruit is among the most abundant metabolites both in MVs and juice. More recently, myo-inositol was also used as an adjuvant for conventional chemotherapy due to its proven anti-tumorigenic properties [35]. Quinic acid, a well-known nutraceutical and anticancer drug candidate in clinical phase trial was also identified among the highly abundant components in the grapefruit juice and MVs samples. Beside quinic acid, oxalic acid is one of the most abundant organic acids present in MVs.

**Figure 4:**
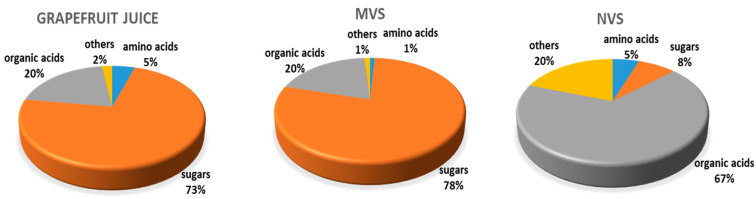
Distribution of main metabolite classes identified in grapefruit juice, and microvesicle and nanovesicle samples. Nanovesicles exhibit a high relative amount of amino acids and organic acids, while microvesicles and fruit juice are characterized by a high percentage of carbohydrates and derivatives.

Figure 4 shows how the common classes of organic compounds, i.e., amino acids and derivatives, carbohydrates and derivatives, organic acids and other metabolites are distributed in the three kinds of samples. Based on this, NVs have a considerably different metabolome profile then whole juice and MVs, while MVs and whole juice are similar to each other. In contrast to the characteristically high sugar content observed in juice (73%) and MVs (78%), NV are characterized by the presence high relative amount of organic acids (67%). Highly expressed organic acids in NVs were alpha-hydroxy acids (AHAs) represented by mainly glycolic and citric acids. Regarding amino acids, while in the juice we could detect a broad range of amino acids including asparagine, aspartic acid, alanine, glutamic acid, leucine, proline, threonine, valine, serine, etc., the amino acid content of MVs and NVs is restricted prevalently to leucine/isoleucine and aspartic acid, this later is present only in MVs (Table S2). Notably, grapefruit-derived MVs contain also aucubin, an iridoid glycoside which is shown to have a variety of pharmacological effects including antimicrobial and anti-inflammatory effects [36].

## 4. Discussion

Citrus fruits have long been used in the traditional medicine of several Asian countries to ameliorate indigestion, cough, skin inflammation and cardiovascular dysfunctions [37]. Phytochemicals derived from citrus fruits show several bioactivities such as anti-oxidative, anti-inflammatory, anti-cancer, as well as cardiovascular- and neuroprotective effects. Interestingly, large epidemiological studies confirmed that regular intake of citrus fruits could prevent many diseases, including cancer [38,39]. Here, by building on the proven beneficial health properties of citrus fruits we have analysed the vesicles enriched fractions isolated from the juice of lemon, grapefruit, bitter and sweet oranges. Our previous study has shown that these samples were enriched in micro- and nano-sized, intra- and extracellular [12] vesicles. Indeed, because of their intrinsic characteristics and role in cellular storage and transport, plant NVs and MVs are extensively exploited for their biological activities and as a platform for loading and delivery of exogenous therapeutics [10]. Despite their promising therapeutic properties, their metabolite cargo remains largely unknown.

In particular, here we showed that lemon, grapefruit, sweet and bitter oranges juice-derived NVs and MVs impair the proliferation of breast, lung and melanoma cells but not Human keratinocyte, HaCaT. These results are in line with the antitumour effects previously reported by Raimondo and colleagues, who demonstrated that nanosized vesicles purified from *C. lemon* inhibit cancer cell proliferation in different tumour cell lines as well as in vivo [11].

We further investigated the biological effects of grapefruit-derived vesicles on A375 melanoma cancer cells at the molecular level. Melanoma is the most aggressive and deadly form of skin cancer. The incidence of melanoma is growing worldwide more than other cancers and the treatment in advanced stages becomes very difficult as metastatic lesions of melanoma are resistant to diverse conventional therapies [40,41].

Anticancer compounds may exert their function through distinct mechanisms acting on DNA replication, cell cycle, migration and apoptosis. Dysregulation of DNA damage repair and signalling to cell cycle checkpoints is recurrent in cancer cells and represent a promising therapeutic target [5]. Our data prove that NVs and MVs from grapefruit juice suppress G0/G1 and G2/M cell cycle transitions, promote the upregulation of the cell cycle inhibitor p21, and reduce the protein expression of Cyclin B1 and the transcript levels of Cyclin B2, key modulators of G2/M checkpoint.

Overall, G2 checkpoint allows the cell to repair DNA damage before entering mitosis. These pieces of evidence suggest that NVs and MVs could be considered as useful tools to target cell cycle checkpoints and control cell proliferation.

Apoptosis maintains proper tissue homeostasis in physiological conditions. Often a loss of balance between cell division and cell death occurs in cancer cells. Therefore, current cancer therapies aim at inducing apoptosis [11]. One of the upstream processes of programmed cell death apoptosis is the activation of caspases, a highly specialized family of cysteinyl-aspartate proteases and the subsequent cleavage of PARP-1 [42]. In this work, we show that, besides inhibition of cell cycle, grapefruit-derived NVs and MVs also trigger apoptosis through the activation of PARP-1, thus further contributing to limit cancer proliferation in vitro.

To identify the compounds responsible for vesicle bioactivity, here we reported the metabolome profiles of grapefruits derived vesicles and compared them with that of juice. GC-MS-based metabolomics study reveals distinct profiles for the juice and vesicle fractions (Figure 3 and Figure 4). Especially, NVs contain fewer carbohydrates and are enriched in alpha hydroxy acids (AHAs, i.e., glycolic and citric acids), amino acids (leucine and isoleucine) and fatty acids (palmitic acid and doconexent) (Table 1 and Table S2). AHAs are a class of chemical compounds that have a carboxylic acid substituted with a hydroxyl group on the adjacent carbon. AHAs are well known for their effect on keratinization and general benefits on skincare and thus their use in the cosmetics industry. In contrast to the high amino acid diversities (i.e., alanine, glycine, valine, leucine/isoleucine, proline, serine, threonine, aspartic acid, asparagine, ornithine, glutamic acid, glutamine phenylalanine, methylalanine and canavanine) of grapefruit juice (Table S2, in the vesicles samples prominently leucine/isoleucine present. Interestingly, the leucine-rich diet was shown to induce a shift in tumour metabolism from glycolytic towards oxidative phosphorylation, reducing thus the glucose consumption and metastasis in rat models [43].

We found the presence of myo-inositol (in all samples) and quininic acid (in MVs and juice) which have shown a significant effect in the treatment of several human diseases [44]. Myo inositol is a carbocyclic sugar with a broad-spectrum anticancer activity. Very interestingly, inositols have been shown to reduce cell proliferation and induce apoptosis by suppressing Akt phosphorylation in prostate and colon cancer [45,46]. Quininic acid, on the other hand, is a crystalline acid found in cinchona bark, coffee beans and many fruits such as apple, berries and citrus. Quininic acid was shown to significantly suppress the invasion of a rat ascites hepatoma in vitro [47] and promoted apoptosis in oral cancer cells by downregulating the expression of anti-apoptotic genes and attenuating the expression of cyclin D1 and Akt signalling pathway [48]. Taken together these data suggest that both myo-inositol and quininic acid delivered by grapefruit vesicles may target Akt signalling in melanoma cells and fuel their anticancer properties.

Remarkably, we found aucubin and doconexent in grapefruit MVs. Aucubin is known for its antimicrobial, anti-inflammatory and anticancer activities [36,49,50]. In particular, aucubin isolated from *Aucuba japonica* was shown to inhibit the proliferation of non-small cell lung cancer A549 cells by blocking cell cycle progression in the G0/G1 phase and inducing apoptosis [51]. Additionally, aucubin promotes dermal wound healing and play a photoprotective role against oxidative stress by inhibiting UVB-induced free radical production in human skin fibroblasts [52,53]. Doconexent, on the other hand, also known as docosahexaenoic acid (DHA), an omega-3 fatty acid has been shown to impair the proliferation of many cancer cell type in vitro and in vivo [54]. In particular. DHA blocks cell cycle progression in the G1 phase and/or in G2M in leukaemia, colorectal, pancreatic, neuroblastoma and breast cancer cells [55]. Interestingly, DHA has also been proved to selectively inhibit melanoma cell growth in vitro and in vivo.

Our study represents a step forward in the analysis of metabolomics content of EV-like vesicles isolated from plants and it shows that citrus vesicles can be a rich source of lead molecules in drug discovery based on their capability to store and transport unique and diverse chemical structures. Indeed, natural nanovesicles transport also lipids, mRNAs, microRNAs, and proteins into the receiving cells, most of them may contribute to their biological activity [56]. Indeed, integrative approaches aiming at the thorough characterization of vesicle cargo are highly recommended to elucidate deeper the biological implications of plant-derived vesicles in nutraceutical and pharmaceutical fields.

In conclusion, our findings reveal that citrus-derived vesicle transport metabolite cargo with therapeutic interest. The vesicular structures could enhance the delivery of labile bioactive molecules through the protection of lipid bilayer as surrounding structure, as well as their efficient and targeted uptake by recipient cells. We also show that vesicles from grapefruit juice impair the growth of diverse tumour cell lines, without affecting normal human keratinocytes cells. Moreover, our data prove that grapefruit-derived NVs and MVs induces G2/M cell cycle arrests, promote apoptosis, and hamper the expression of various critical hallmarks of melanoma, such as those involved in cell growth and proliferation, invasiveness and migration. Although further investigations on in vivo pre-clinical and clinical models are needed, these data point out that citrus-derived vesicles could be excellent and safe biomaterial in their native forms and thus may be considered as co-adjuvants for treatment of human malignancies.

## Figures and Tables

**Table 1:** Top ranking metabolites tentatively-identified in grapefruit (**A**) fruit juice, (**B**) MVs and (**C**) NVs.

**A**		
**N°**	**Area Counts × Min**	**Name**
1	8.4 × 10^8^	D-(−)-Fructose, pentakis(trimethylsilyl) ether, methyloxime (syn)
2	4.2 × 10^8^	Citric acid, 4TMS derivative
3	4.2 × 10^8^	d-Glucose, 2,3,4,5,6-pentakis-O-(trimethylsilyl)-, o-methyloxyme, (1Z)-
4	3.7 × 10^8^	Sucrose, 8TMS derivative
5	2.7 × 10^8^	Myo-Inositol, 6TMS derivative
6	1.7 × 10^8^	d-Galactose, 2,3,4,5,6-pentakis-O-(trimethylsilyl)-, o-methyloxyme, (1Z)-
7	6.5 × 10^7^	Lactulose, octakis(trimethylsilyl) ether, methyloxime (isomer 1)
8	6.2 × 10^7^	Quininic acid (5TMS)
9	4.8 × 10^7^	L-Aspartic acid, 2TMS derivative+L-Aspartic acid, 3TMS derivative
10	3.1 × 10^7^	Malic acid, 3TMS derivative
11	2.3 × 10^7^	Serine, 3TMS derivative
12	2.1 × 10^7^	Glycolic acid, 2TMS derivative
13	1.9 × 10^7^	L-Proline, 2TMS derivative
14	1.7 × 10^7^	d-Ribose, 2,3,4,5-tetrakis-O-(trimethylsilyl)-, O-methyloxime
15	1.6 × 10^7^	4-Aminobutanoic acid, 3TMS derivative
16	1.4 × 10^7^	N-Acetyl glucosamine methoxime, tetrakis(trimethylsilyl)
17	1.3 × 10^7^	D-(+)-Turanose, octakis(trimethylsilyl) ether
18	9.9 × 10^6^	L-Alanine, 2TMS derivative
19	9.1 × 10^6^	L-Glutamic acid, 3TMS derivative
20	7.2 × 10^6^	α-D-Glucopyranoside, methyl 2-(acetylamino)-2-deoxy-3-O-(trimethylsilyl)-, cyclic methylboronate
**B**		
**N°**	**Area Counts × Min**	**Name**
1	6.0 × 10^8^	D-Fructose, 1,3,4,5,6-pentakis-O-(trimethylsilyl)-, O-methyloxime
2	4.1 × 10^8^	Sucrose, 8TMS derivative
3	3.7 × 10^8^	d-Glucose, 2,3,4,5,6-pentakis-O-(trimethylsilyl)-, o-methyloxyme, (1Z)-
4	2.9 × 10^8^	Citric acid, 3TMS derivative+Citric acid, 4TMS derivative
5	2.1 × 10^8^	Myo-Inositol, 6TMS derivative
6	1.7 × 10^8^	d-Galactose, 2,3,4,5,6-pentakis-O-(trimethylsilyl)-, o-methyloxyme, (1Z)-
7	1.4 × 10^8^	Oxalic acid, 2TMS derivative
8	6.9 × 10^7^	D-(+)-Turanose, octakis(trimethylsilyl) ether
9	4.0 × 10^7^	Quininic acid (5TMS)
10	3.9 × 10^7^	β-D-Galactopyranoside, methyl 2,3-bis-O-(trimethylsilyl)-, cyclic methylboronate
11	3.4 × 10^7^	α-D-Glucopyranoside, methyl 2-(acetylamino)-2-deoxy-3-O-(trimethylsilyl)-, cyclic methylboronate
12	3.2 × 10^7^	Lactulose, octakis(trimethylsilyl) ether (isomer 1)
13	2.8 × 10^7^	(2S,3R)-3-[(4E,7E)-Nona-4,7-dienoyl]-N,N-bis(trimethylsilyl)oxirane-2-carboxamide
14	2.1 × 10^7^	N-Acetyl glucosamine methoxime, tetrakis(trimethylsilyl)
15	2.0 × 10^7^	D-Mannitol, 6TMS derivative
16	1.6 × 10^7^	D-(−)-Tagatose, pentakis(trimethylsilyl) ether, methyloxime (anti)
17	1.5 × 10^7^	L-Aspartic acid, 2TMS derivative+L-Aspartic acid, 3TMS derivative
18	1.4 × 10^7^	Aucubin, hexakis(trimethylsilyl) ether
19	1.0 × 10^6^	Malic acid, 3TMS derivative
20	8.1 × 10^6^	Myo-Inositol, pentakis-O-(trimethylsilyl)-, bis(trimethylsilyl) phosphate
**C**		
**N°**	**Area Counts × Min**	**Name**
1	1.1 × 10^8^	Glycolic acid, 2TMS derivative
2	3.1 × 10^7^	(2S,3R)-3-[(4E,7E)-Nona-4,7-dienoyl]-N,N-bis(trimethylsilyl)oxirane-2-carboxamide
3	4.7 × 10^7^	L-Isoleucine, 2TMS derivative
4	4.3 × 10^6^	L-Leucine, TMS derivative+Leucine, 2TMS derivative
5	3.9 × 10^6^	d-Glucose, 2,3,4,5,6-pentakis-O-(trimethylsilyl)-, o-methyloxyme, (1Z)-
6	3.6 × 10^6^	D-(−)-Tagatose, pentakis(trimethylsilyl) ether, methyloxime (anti)
7	2.1 × 10^6^	D-Psicose, pentakis(trimethylsilyl) ether, methyloxime (syn)
8	1.3 × 10^6^	Urea, 2TMS derivative
9	1.2 × 10^6^	D-(+)-Turanose, octakis(trimethylsilyl) ether
10	1.1 × 10^6^	Lactic Acid, 2TMS derivative
11	9.4 × 10^5^	d-Mannose, 2,3,4,5,6-pentakis-O-(trimethylsilyl)-, o-methyloxyme, (1Z)-
12	7.6 × 10^5^	L-(−)-Sorbose, pentakis(trimethylsilyl) ether, methyloxime (anti)
13	5.9 × 10^5^	Citric acid, 4TMS derivative
14	4.5 × 10^5^	Sucrose, 8TMS derivative
15	1.5 × 10^5^	Myo-Inositol, 6TMS derivative
16	1.3 × 10^5^	Palmitic Acid, TMS derivative
17	7.5 × 10^4^	2-Hydroxycyclohexane-1-carboxylic acid, bis(trimethylsilyl) deriv.
18	4.7 × 10^4^	Doconexent, TMS derivative

## Supplementary Materials

The following are available online at https://www.mdpi.com/2073-4409/9/12/2722/s1, Table S1: List of primers employed in qRT-PCR analyses., Table S2: Metabolites of (A) grapefruit juice, (B) microvesicles and (C) nanovesicles tentatively identified by GC-MS List of primers employed in qRT-PCR analyses.
